# Can testing of six individual muscles represent a screening approach to upper limb neuropathic conditions?

**DOI:** 10.1186/1471-2377-14-90

**Published:** 2014-04-26

**Authors:** Jørgen Riis Jepsen

**Affiliations:** 1 Department of Occupational Medicine, Hospital of South-western Jutland, Østergade 81-83, Esbjerg DK-6700, Denmark; 2 Centre of Maritime Health and Society, University of Southern Denmark, Niels Bohrs Vej 9-10, Esbjerg DK-6700, Denmark

## Abstract

**Background:**

It has previously been demonstrated that an extensive upper limb neurological examination of individual muscle function, sensation in homonymous innervated territories, and nerve trunk allodynia is reliable and that the outcome reflects symptoms. Since this approach may appear complicated and time consuming, this study deals with the value of an examination limited to manual testing of only six muscles.

**Methods:**

Two examiners blinded to symptom status performed manual muscle testing of six muscles in 82 upper limbs with or without pain, weakness, and/or numbness/tingling. The six muscles represent three antagonist pairs (pectoralis major/posterior deltoid, biceps/triceps, and radial flexor of wrist/short radial extensor of wrist). The inter-rater reliability of detecting muscular weaknesses and the relation of weakness to the mentioned symptoms were analysed by kappa-statistics.

**Results:**

The two examiners recognized weaknesses in 48 and 55 limbs, respectively, with moderate agreement (median kappa = 0.58). Out of these, 35 and 32 limbs, respectively, were symptomatic. There was good correlation between findings and symptoms for one examiner (kappa = 0.61) and fair correlation for the other one (kappa = 0.33). Both reached high sensitivity (0.92, 0.84) but less satisfactory specificity (0.70, 0.50). Weaknesses agreed upon by the two examiners correlated moderately with symptoms (kappa = 0.57).

**Conclusions:**

Weakness in one or more muscles was present in almost all symptomatic limbs but in many non-symptomatic limbs as well. Manual testing of six muscles may represent a useful screening approach to upper limb neuropathic conditions, but a confirmative diagnosis requires further assessment.

## Background

The high prevalence of work-related upper limb disorders, their effects on the quality of life and work capacity, and the limited progress concerning their diagnoses, management and prevention require new perspectives in this field of research and practice. In particular, there is a clear need of consensus regarding physical tests and diagnostic criteria of sufficient diagnostic efficacy [1,2].

Palmer and Cooper estimated that a standard physical approach permits diagnostic classification of only a quarter of patients with work-related upper limb disorders [3]. The remaining patients are frequently regarded as suffering from so-called “non-specific” conditions that may be labeled, e.g. “repetition strain syndrome” indicating a state of turbid pathology, which is assumed to be related to adverse physical work exposures. Even in the absence of supporting evidence in terms of physical findings, however, there is also a tendency to diagnose many patients with upper limb disorders according to the dominant location of symptoms, e.g. as epicondylitis with elbow pain or rotator cuff disorder with shoulder pain. Such diagnostics, however, neither reflect the type of the injured tissue, its location nor the implicated pathology. If symptoms are perceived as of a neuropathic nature, these patients are likely to be subjected to electrophysiological or imaging studies rather than a thorough physical upper limb examination of the peripheral nerve-functions.

The value of case definitions (diagnostic criteria consisting of a combination of symptoms and signs that characterize a certain disorder) lies in their practical utility in distinguishing groups of people with the same symptoms and/or physical characteristics, or whose illness share the same causes or determinants of outcome. Therefore the best diagnostic case definition of a disorder may vary according to the purpose, e.g. epidemiological or clinical, for which it is being applied [4]. Still, the case definition should reflect the injured tissue and its location.

Many patients with upper limb disorders present a triad of symptoms consisting of weakness, numbness/tingling and pain, which is frequently of a neuropathic character. These symptoms suggest a peripheral nerve-involvement. Patterns of neurological abnormalities defined from the course and innervation of nerves (selective muscle weakness [5], sensory abnormalities and mechanical nerve trunk allodynia) may reflect upper limb focal neuropathy with specific locations.

This has resulted in the development and validation of a rather extensive systematic and detailed neurological upper limb assessment, with the intention of complementing the standard physical approach to patients referred to a department of occupational medicine. The neurological examination is based on the likeliness of an impairment of the motor and sensory functions distal to an entrapped segment of a supplying nerve, and on the nerve being abnormally sore on palpation at the location of entrapment. We have demonstrated that this examination is reliable and able to discover the presence of neurological patterns in symptomatic upper limbs and their absence in non-symptomatic limbs [6-8]. Consequently, this neurological assessment may be important, in particular when the standard physical examination fails to identify abnormalities. This was the case in 13 out of 16 symptomatic limbs that could otherwise not be characterized diagnostically, i.e. “non-specific arm pain” [8].

Clinicians who are less familiar with the biomechanical properties of the muscles and with the course of upper limb nerves and their muscular and cutaneous innervation may regard such an extensive neurological examination [6,7] as time consuming, and difficult to perform and interpret. Therefore, a simple screening approach to the upper limb nerves would be of significance. In the 1993 meeting in The Scandinavian Society for Surgery of the Hand, Hagert presented an examination based on manual muscle testing of six muscles that were selected out of 60 shoulder and upper limb muscles (pectoralis major/posterior deltoid, biceps/triceps, and radial flexor of wrist/short radial extensor of wrist). This examination was developed to reflect focal neuropathy with specific locations. He concluded that an affliction of the upper limb nerve tree was unlikely with normal strength in these muscles as well as in the small abductor to the 5th digit, the small abductor of the thumb, and the ulnar extensor of the wrist [9]. A more recent publication provides a detailed description of the technique of the testing of eight muscles that are representative to the upper limb nerves and the interpretation of the outcome [5].

**Figure 1:**
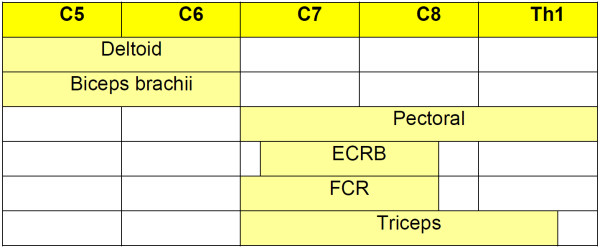
**Roots, brachial plexus, and upper limb peripheral nerves.** Overview and innervation of the selected six upper limb muscles.

The six muscles suggested by Hagert (Table 1) were selected for this study because they are simple to remember and examine. In addition, they are reasonably representative of four (C5, C6, C7 and C8) out of the five cervical roots forming the brachial plexus (Figure 1) as well as of the brachial plexus and most individual upper limb nerves. Consequently, one or more of these muscles are likely to be involved with many upper limb nerve afflictions. Weakness in these muscles may reflect peripheral focal neuropathy and, according to experience, is a common finding if looked for.

**Table 1:** Manual testing of three muscle antagonist pairs in 82 upper limbs

**Muscle antagonist pair**	**Muscle**	**Nerve**	**Weakness**		**Relative agreement% [**[6]**]**	**Kappa-value (Confidence intervals) [**[6]**]**	**Exit position for muscle testing [**[6]**]**
			**Examiner 1**	**Examiner 2**			
**I**	Greater pectorals	Pectorals	16	21	84	0.55 (0.34-0.76)	90 degrees shoulder flexion. Upper extremities placed horizontally forward, forearms pronated (Figures 2 and 3)
	Posterior deltoid	Axillary	48	50	80	0.59 (0.42-0.77)	
**II**	Biceps brachii	Musculocutaneous	36	31	79	0.57 (0.40-0.75)	90 degrees elbow flexion. Upper arms placed vertically against the lateral chest and forearms horizontally. The supinator function of the biceps may additionally be tested (Figures 4 and 5)
	Triceps	Radial	34	33	87	0.72 (0.57-0.88)	
**III**	Radial flexor of wrist	Median	32	32	77	0.46 (0.25-0.66)	90 degrees elbow flexion. Forearms resting fully on thighs: For the testing of the radial flexor of wrist, forearms are supinated and fingers flexed. For the testing of the short radial extensor of wrist, forearms are pronated and fingers extended (Figures 6 and 7)
	Short radial extensor of wrist	Radial	29	20	84	0.69 (0.53-0.85)	

This study aims at clarifying two questions, which are crucial for the assessment of how far a limited examination is a feasible initial physical approach to the upper limb nerves:

! Is it possible to reliably identify weakness in the selected six upper limb muscles?

! Does the presence of weakness in any of these six muscles correlate to the patients’ complaints in terms of pain, weakness, and/or numbness/tingling?

## Methods

### Patients

The participating patients were identical to those in the previously studied series of 41 consecutively referred patients: 22 males of median age 44 years (range 29–61), and 19 females of median age 39 years (range 25–52). Prior to the examination the study-patients were selected according to defined criteria among patients referred with any disorder (whether or not confined to the upper limbs) to the Department of Occupational Medicine, Hospital of South-western Jutland Esbjerg. Seventeen patients were excluded because they were known from earlier contacts, had problems concerning communication, had undergone previous upper limb surgery, or had an appearance suggesting easily recognizable disease such as severe asthma or disabling low back disease. Fifteen patients refused participation. For capacity reasons (max. one study patient/day), ten patients comparable to the study patients with respect to disease pattern and severity were additionally excluded.

Twenty-two patients were referred due to complaints from one limb and five patients due to complaints from both upper limbs. Out of nine patients referred for reasons unrelated to upper limb complaints six patients had present complaints and three patients have had previous upper limb complaints. Five patients had had no current or previous upper limb complaints [6-8]. The patients represented a broad spectrum of disease with regard to severity and duration. They were preferentially referred for assessment of the potential work-relatedness of their disorder and the consequences of their disorder for their future working life. Patients with upper limb complaints were referred with unspecific diagnoses or their complaints were interpreted as being related to conditions such as rotator cuff disorder, epicondylitis or carpal tunnel syndrome. Thus their symptoms might or might not be related to a neurological disorder. The referral diagnoses of patients that were not referred due to upper limb complaints were, e.g. dermatitis or asthma.

The study complied with the Helsinki declaration. It was approved by the Ethics Committee (De Videnskabsetiske Komitéer for Region Syddanmark) and signed informed consent was obtained from all participants.

### Methods

#### Inter-rater reliability

All patients underwent identical neurological bedside assessments by two examiners. No communication occurred between the two examiners. The examinations took place in separate examination rooms and were performed in immediate succession one after the other. Both examiners were completely blinded to any patient characteristics, and communication with the patients was limited to instructions with regard to the examination. While the examination comprised the items previously reported (14 individual muscles, as well as sensibility and mechanical nerve trunk allodynia at defined locations [6,7], the current study only assessed the outcome of the manual testing of six individual upper limb muscles (on both sides) representing three pairs of antagonists (Table 1).

Technical skills in the manual muscle strength testing procedure are crucial. Some experience is needed in performing the examination correctly, and in interpreting strength as normal or reduced. Slightly reduced strength may be particularly difficult to assess. Both examiners had learned the examination technique a short time before the study. One examiner had used it for two years prior to the study and the other one for only two months following an update on upper limb anatomy and supervised examination of about 20 patients.

#### Procedure for the manual semi-quantitative isometric muscle testing

Each antagonist pair of muscles was examined strictly systematically from the two proximal muscles to the two distal muscles. The right and the left side was assessed simultaneously with the limb placed in a position that optimizes the isolated function of the particular muscles examined (Table 1) [6,10].

**Figure 2:**
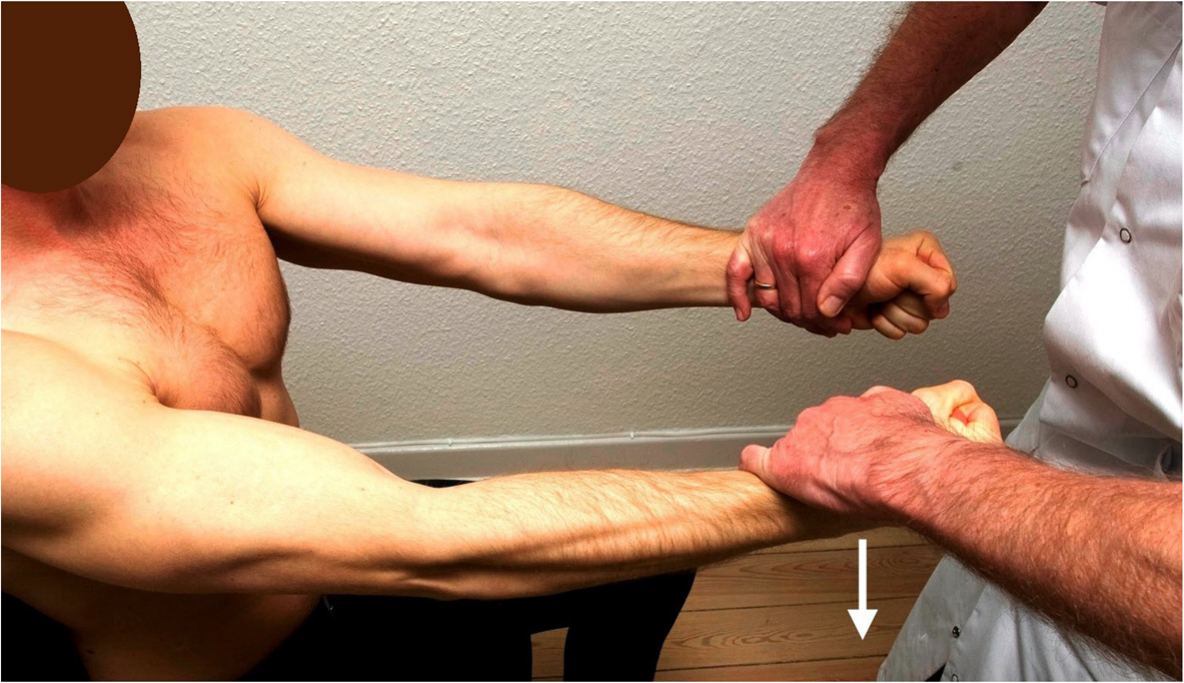
**Standard posture I.** Testing of the pectoral muscle. The arrow illustrates the direction of the examiner’s force against the patient’s resistance. The posterior deltoid muscle works as the antagonist.

**Figure 3:**
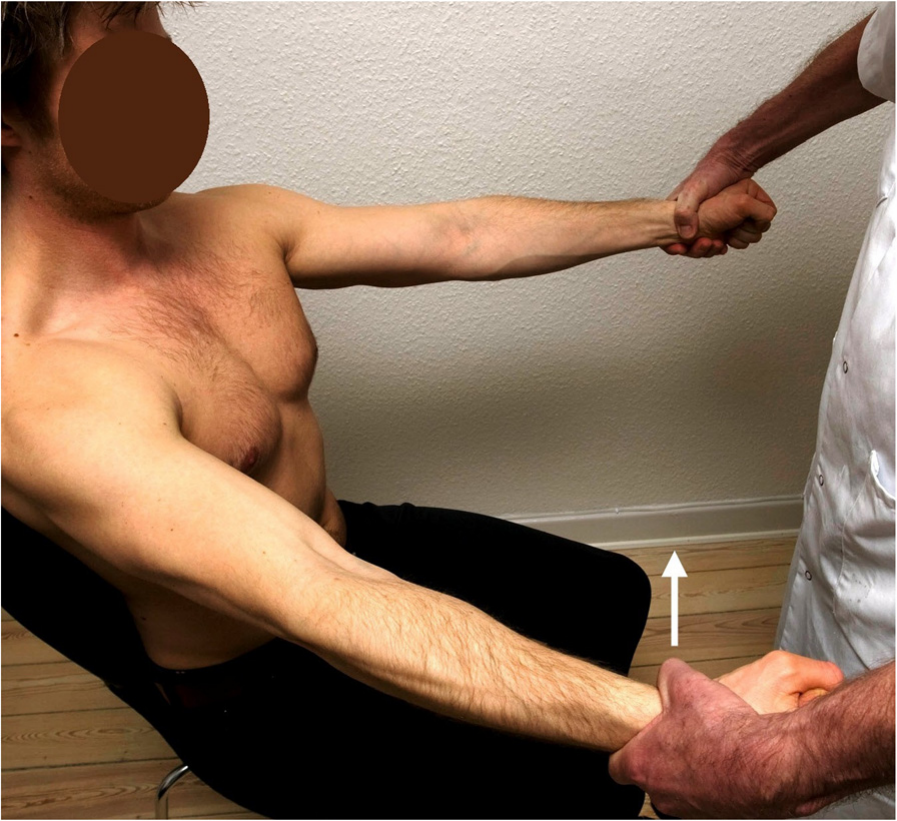
**Standard posture I.** Testing of the posterior deltoid muscle. The arrow illustrates the direction of the examiner’s force against the patient’s resistance. The pectoral muscle works as the antagonist.

**Figure 4:**
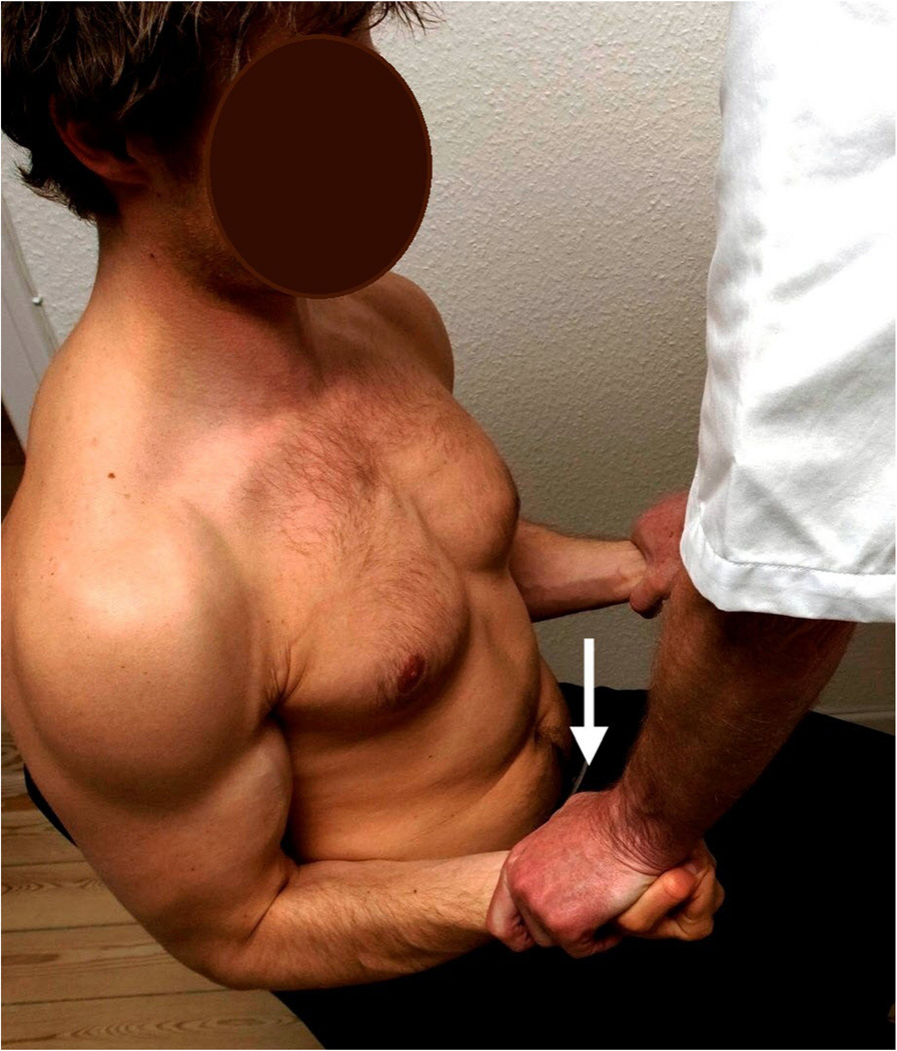
**Standard posture II.** Testing of the biceps brachii muscle. The arrow illustrates the direction of the examiner’s force against the patient’s resistance. The triceps muscle works as the antagonist.

**Figure 5:**
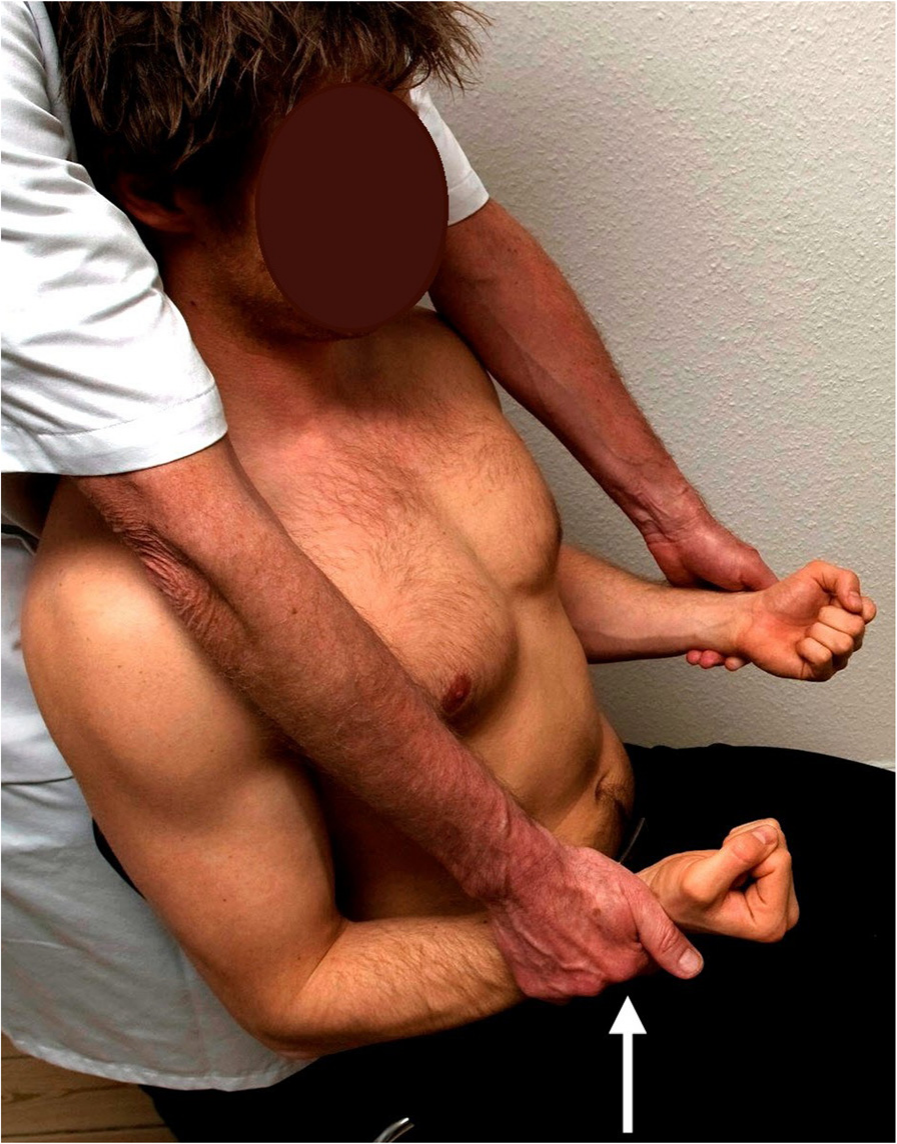
**Standard posture II.** Testing of the triceps muscle. The arrow illustrates the direction of the examiner’s force against the patient’s resistance. The biceps brachii muscle works as the antagonist.

**Figure 6:**
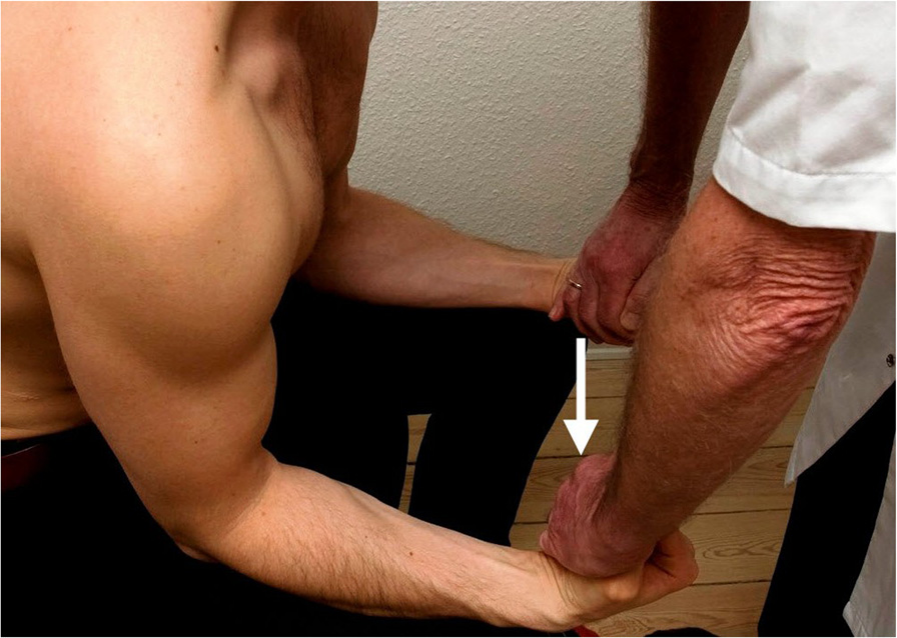
**Standard posture III.** Testing of the flexor carpi radialis muscle. The arrow illustrates the direction of the examiner’s force against the patient’s resistance. The short extensor of wrist muscle works as the antagonist.

**Figure 7:**
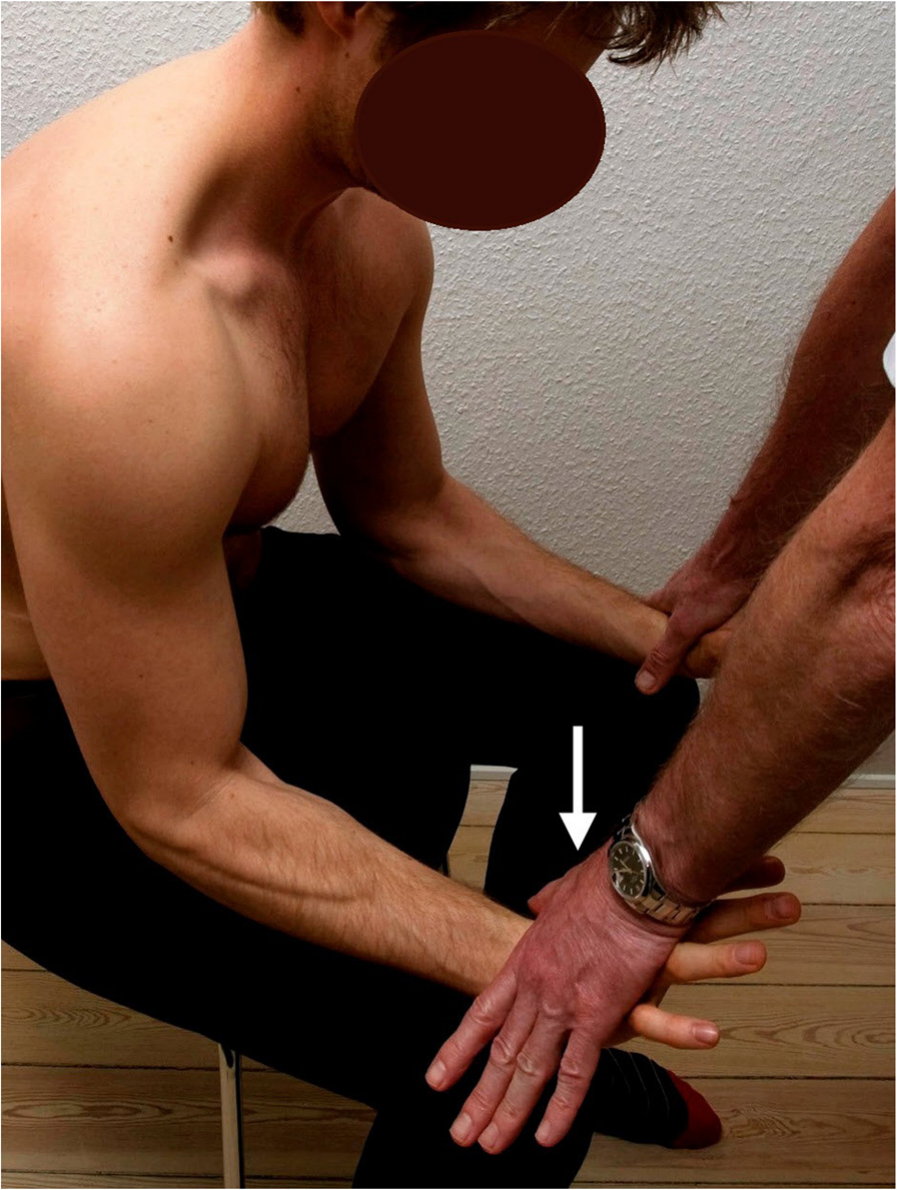
**Standard posture III.** Testing of the extensor carpi radialis brevis muscle. The arrow illustrates the direction of the examiner’s force against the patient’s resistance. The flexor carpi radialis muscle works as the antagonist.

I. The patient’s arms were elevated horizontally forward, with the elbows kept fully extended, the forearms pronated, the wrists kept at neutral and the hand clenched. With the examiner standing in front of the patient, the arm adduction (pectoral muscles) and abduction (posterior deltoid) were tested by applying force against the patient’s wrists from inward-out and from outward-in, respectively (Figures 2 and 3, respectively). The preferred exit position for the posterior deltoid is to have the patient keep the arms 30 degrees outward.

II. The patient’s upper arms were now kept along the sides of the chest, the elbows flexed at a right angle and propped against the chair back, the forearms pointing forward and kept at neutral position, the wrists kept at neutral and the hands clenched. Standing in front of the patient, the examiner leaned forward toward the patient’s wrists, asking the patient to “carry” the examiner (elbow flexion, defined as biceps) (Figure 4). Finally, standing behind the patient, the examiner lifted the patient’s wrists upward (triceps) against the patient’s resistance (Figure 5).

III. The patient leaned forward, resting the forearms on the thighs with the wrists just distal to the knees. With the patient’s forearms fully supinated, hands clenched and the wrists slightly flexed, the examiner leaned forward, pressing toward the proximal interphalangeal joint knuckles of the index and long fingers to extend the wrists of the patient (FCR) (Figure 6). With the patient’s forearms fully pronated, the hands kept open and the wrists extended, the examiner leaned forward, pressing against the knuckles of the index and long fingers to flex the patient’s wrists (ECRB) (Figure 7).

Several classification systems may be applied for the assessment of muscle function. This study as well as the Motricity Index and the Motor Index Score use the grading system of the Medical Research Council [11], and all these classification systems seek to characterize status using a limited number of muscles.

Both examiners classified each limb with respect to either the presence of weaknesses defined as grade 4+ or less in any of the six muscles in Table 1 or to intact strength in all six muscles.

#### Construct validity

The construct validity of the examination can be studied by examining whether measures of constructs that theoretically should or should not be related to each other are, in fact, related to each other. Muscular weakness, which is caused by a neurologic affliction such as nerve entrapment, is likely to be symptomatic (convergent validity), while symptoms are less likely in limbs without weakness (discriminant validity). Therefore the presence or absence of weakness(es) was compared to the presence or absence of upper limb complaints (pain, weakness and/or numbness/tingling).

Information on the patients’ upper limb complaints was collected by two interviewers who did not communicate with the examiners mentioned above [8].

### Statistics

The agreement between the examiners in terms of identifying individual muscle weakness(es) and pattern(s) of weakness was assessed by Cohen’s kappa-statistics. The same statistics were employed for estimating the relation of presence of any pattern(s) to the standard criterion (pain, weakness and/or numbness/tingling).

Cohen’s 6-statistics is a measure for testing whether agreement between raters of categorical data exceeds chance levels: kappa = (p_o_ - p_e_)/(1 - p_e_) where p_o_ is the proportion of observed agreement; and p_e_ is the proportion of agreement expected by chance. The kappa-coefficient has a maximum of 1.0 and is interpreted as kappa: < 0.2 = poor, 0.21-0.40 = fair, 0.41-0.60 = moderate, 0.61-0.80 = good, 0.81-1.00 = very good [12].

In addition, we calculated the sensitivity and specificity of the approach in terms of the ability of identified weakness to predict the presence of symptoms.

## Results

### Inter-rater reliability

Manual testing of each of the six selected individual muscles was reliable. The median relative agreement and the median kappa-value for the six individual muscles were 82% (range 77-87%) and 0.58 (range 0.46-0.72), respectively (Table 1).

The two examiners identified weakness in one or more muscles in 48 and 54 limbs, respectively, with agreement on the presence or absence of any weakness in 43 and 23 limbs, respectively, and disagreement in 16 limbs (Table 2). The resulting inter-rater reliability was moderate (80% relative agreement, kappa = 0.59, CI 0.44-0.77).

**Table 2:** Inter-rater reliability of the identification by the two examiners of any weakness in six muscles

		**Examiner 2**		**Total**
	**Any weakness**	**Absent**	**Present**	
**Examiner 1**	**Absent**	**23**	11	34
	**Present**	5	**43**	48
**Total**		28	54	82

### Correlation between the presence of symptoms and the identification of weakness by the two examiners

For one examiner, the examination of the six muscles resulted in a good correlation between the identification of any weakness and the presence of symptoms (kappa = 0.61, CI 0.45-0.78), while the other achieved a fair correlation only (kappa = 0.33, CI 0.13-0.53). The two examiners found weakness in one or more muscles in 35 and 32, respectively, out of 38 symptomatic limbs. No weakness was found in 31 and 22, respectively, out of 44 non-symptomatic limbs.

Consequently, the diagnostic sensitivity of the assessment by each examiner was 0.92 and 0.84, respectively, and the specificity 0.70 and 0.50, respectively. In this sample, the positive/negative predictive values with respect to symptoms were 0.73/0.91, respectively, for one examiner and 0.59/0.79 for the other (Table 3).

**Table 3:** Upper limb symptoms related to the identification of any weakness in six muscles

**Upper limb symptoms**	**Examiner 1**			**Examiner 2**			**Total**
	**No weakness identified**	**Any weakness present**	**Kappa**	**No weakness identified**	**Any weakness present**	**Kappa**	
**Absent**	31	13	0.61	22	22	0.33	44
**Present**	3	35		6	32		38
**Total**	34	48		28	54		82

### Correlation to symptoms with agreement between the two examiners

In 79% of limbs with unanimous rating of presence or absence of weakness by the two examiners, the rating was in agreement with the presence of symptoms and the kappa-value was thus calculated to 0.57 (CI 0.37-0.77. The sensitivity of unanimously concluding the presence of weakness in symptomatic limbs was 0.84, whereas the specificity of identifying absence of weakness in non-symptomatic limbs was just 0.45. The positive and negative predictive values were 0.74 and 0.87, respectively (Table 4).

**Table 4:** Agreement between the identification of any weakness by the two examiners and the relation to symptom status

**Symptoms**	**Agreement on absence**	**Disagreement**	**Agreement on presence**	**Total**
**Absent**	**20 (38)**	13 (4)	11 (2)	44
**Present**	3 (4)	3 (6)	**32 (28)**	38
**Total**	23	16	43	82

The numbers in brackets represent the corresponding figures with application of the full examination [8].

## Discussion

The neurological examination, in particular the assessment of individual muscle function, is disfavored by being assumed by many as “subjective” and consequently not trustworthy. In spite of little evidence for such a standpoint, many and frequently severely affected patients that complain of upper limb pain, numbness and/or weakness are met with skepticism and a tendency to reject a somatic origin of their symptoms [13-16]. This, in particular, may apply for the many patients with work-related upper limb complaints that are unclassifiable according to current examination practices and diagnostic criteria.

The previously presented extensive examination of neurological items selected to represent the function of the upper limb nerves has been shown to be precise *and* able to accurately predict the presence of symptoms [6-8] (Table 4). Although representing a standard approach that should be included in the physical upper limb examination, the neurological part of the examination is rarely applied with a great level of details and may be regarded as complicated to perform and interpret.

There were several reasons for studying the feasibility of limiting an upper limb neurological examination to the assessment of strength in only six upper limb muscles. I was concerned about the obvious diagnostic difficulties that clinicians face when encountering patients with upper limb complaints – in particular patients that cannot be classified according to common diagnostic criteria, and also about the unjustified but widespread use of diagnostic labeling that neither reflects the injured tissue nor its location and pathology. However, I was also concerned about arguing for an extensive neurological examination that clinicians may find too difficult or time consuming.

To meet this challenge, the aim was to present a simple examination of neurological items, which by the identification of neurological signs can complement the standard physical upper limb examination and with a high degree of certainty can contribute to the diagnosis by explaining symptoms that could be due to a nerve-related condition.

Manual testing of only three pairs of antagonist muscles working over the shoulder, the elbow and the wrist is rapid and easy to remember. The interpretation of the outcome of this examination is also relatively simple.

Muscle weakness was frequent in the studied sample of patients and could be identified with a median kappa-value 0.58 (Table 1). This reliability is acceptable and, in fact, superior to that of other parts of the neurological examination usually relied on, e.g. the Babinski sign [17]. The consistency in between the two examiners of their findings argues against a bias of their way of examining the patients. Therefore the testing of these six muscles meets the requirements of simplicity and reliability.

The two examiners found weakness in one or more muscles in 92% and 84% of the 38 symptomatic limbs, respectively, meaning that this limited examination is able to identify weakness in almost all symptomatic limbs in the studied sample. Considerable weakness (20-25%) can be present even when external muscle resistance does not reveal it and therefore a certain amount of reduced strength is required for the detection by manual muscle testing [18]. On this background the presented findings are noteworthy. With full inter-rater agreement, the estimates of the two examiners concerning the presence or absence of weakness reflected the subjective symptoms moderately well (kappa = 0.57). In limbs with agreement between the assessment of the two examiners, the sensitivity of this limited examination was even higher than that of the previously presented extensive examination (0.84 and 0.73, respectively) [8].

The identified weaknesses may be due to neuropathic or non-neuropathic conditions. While this study cannot distinguish between these it does suggest the presence of a nerve involvement in a proportion of the symptomatic limbs with muscle weakness while a nerve involvement is less likely in symptomatic limbs with intact strength in all six muscles. In this sample of patients there were few symptomatic upper limb conditions without a neuropathic component. This observation differs from the general perception of work-related upper limb disorders as mainly located in tendons, insertions, and muscles etc.

The two examiners also identified weaknesses in a high proportion (30% and 50%, respectively) of the 44 non-symptomatic limbs (Table 4). In limbs with agreement between the two examiners, the specificity of the examination limited to six muscles was only 0.45 while the previously presented extensive examination was much more specific (0.86) [8]. The weakness in non-symptomatic limbs cannot be explained but is hardly related to the presence of an ongoing peripheral nerve affliction such as entrapment, which is likely to be painful.

The low specificity indicates that an examination limited to the testing of six muscles is clearly not suitable for confirmative diagnostic purposes, and that the identification of reduced strength requires further examination such as an assessment of nerve trunk allodynia to identify or rule out a nerve affliction.

### The concept of weakness

Weakness can be an objective and/or a subjective phenomenon. It may be of a global character or limited to one or a few muscles, e.g. muscles with shared innervation. Whether muscular weakness is experienced by the patient or not, the objective phenomenon of identifying reduced strength in one or more individual muscles during the physical examination may be subject to interpretation.

Weakness may reflect the state of muscles of a healthy subject in a bad physical condition, e.g. consequent to inactivity, or be related to asthenia accompanying a disorder that may or may not be confined to the musculoskeletal system *per se*. Muscular weakness may also be simulated for the achievement of some advantage. Common to these situations is that weaknesses rarely occur in patterns with some muscles weak and others intact. Furthermore, weakness may be pain-induced (although this term is probably used more than it is justified). Pain-induced weakness tends to involve a single muscle or a few muscles whereas other muscles with the same innervation can usually be tested without pain-aggravation.

Individual weakness or patterns of weakness are not necessarily due to *pareses*, i.e. an affliction of the peripheral (or central) nervous system. Global weakness (in this case in all six examined muscles) is rare, but if present it may represent an affliction of all cords of the brachial plexus, or it may be a result of the causes listed above. Weakness in a single muscle may also have other causes than an affliction of the nerve that innervates that muscle. Painful use of a certain muscle due to a tendinitis, for example, may prevent the exertion of full strength and will be accompanied by soreness of the structure. However, weaknesses of several muscles with a pattern in accordance with anatomical facts (such as innervation patterns of a peripheral nerve) are more likely to represent pareses and reflect a neurological rather than a non-neurological condition. Furthermore, the identification of mechanical allodynia at the location along the nerve trunk where, according to the pattern of weakness a nerve affliction may be located, would suggest the weakness to be related to a focal neuropathic condition.

A pattern of muscular paresis occurring secondary to a peripheral nerve affliction such as entrapment is likely to be accompanied by mechanical nerve trunk allodynia at the site of entrapment [7]. The absence of nerve trunk soreness, on the other hand, argues against the theory that ongoing nerve entrapment is causing the weakness. In the same way, muscular weakness accompanied by impaired sensation is more likely to represent a paresis, if the weak muscle and the skin with impaired sensation have the same innervation. Consequently, the demonstration of nerve trunk allodynia or sensory dysfunction, with an appropriate location in addition to weakness, will increase the specificity compared to that of the isolated examination of muscle strength.

In the previous study of the same sample of patients, there was a remarkable tenderness of nerve trunks at specific locations that were related to the discovered patterns of weakness [7]. This finding suggests that the identified weaknesses represent pareses. E.g. mechanical allodynia was present at the brachial plexus in the deltoid-pectoral groove in all 14 limbs in which both examiners identified a pattern of weakness (posterior deltoid, biceps, flexor carpi of the wrist) in accordance with an infraclavicular brachial plexopathy (pectoralis minor syndrome) [7]. Therefore, the assessment of nerve trunk allodynia in addition to muscle strength testing would improve specificity.

### Standard for comparison

Pareses would most likely be symptomatic (convergent validity), while symptoms would be relatively less probable in limbs without pareses (discriminant validity). This logic applies despite the recognition that symptoms may have other causes than nerve affliction(s), and that nerve afflictions may be non-symptomatic. While it is therefore acknowledged that symptoms are not an ideal standard for comparison, it is, however, not possible to apply a better standard in this study. A gold standard does not exist [8].

For electrophysiology to serve as gold standard it would require a global assessment of nerve conduction at many levels in a large number of nerves as well as electromyographic studies of multiple muscles. Such an extensive bilateral examination of the upper limb nerves, the brachial plexus and the roots would be painful, expensive and very time consuming, and just for that reason not feasible. More importantly, however, electrophysiology is subject to interpretation and for technical and other reasons cannot serve as a valid estimate for focal neuropathy [19]. E.g. median nerve compression at the elbow level (pronator syndrome) can rarely be detected by the measurement of motor or sensory conduction velocity [20,21]. The electrophysiological diagnosis of radial tunnel syndrome is also unreliable [22]. Brachial plexopathy constitutes a major challenge with regard to electrodiagnosis [23-26]. Unfounded confidence to the electrophysiological assessment of nerve entrapments such as these may prevent a correct diagnosis by failing to emphasize physical neurological parameters such as those applied in this and previous studies [6,7]. Comparable limitations apply for imaging studies. While nerves and surrounding abnormalities can be visualized [27], the accuracy with regard to the detection of e.g. nerve entrapment by ultrasound and MR-imaging remains unknown. This is because the outcome of imaging has been compared with standards such as surgical findings or electrophysiology [28] that cannot serve as valid estimates for these conditions. An attempt to do so may additionally cause a risk of confounding by indication.

### Interpretation

Muscle weakness in accordance with the innervation suggests to the examiner that the patient is trustworthy, whereas occurrence of weakness with a random distribution should be critically scrutinized. Interpretation of the outcome of muscle testing has been provided by Hagert and Hagert [5]. With a normal strength in the six muscles selected for this study, several locations of neuropathy would be unlikely, e.g. the brachial plexus, the axillary nerve, the radial nerve, and the median nerve at elbow level. On the other hand, unless weakness in any of these muscles cannot be satisfactorily explained from other reasoning in a symptomatic limb, the clinician should not uncritically conclude neuropathy to be absent.

The identification of any weak muscle should be followed by a more comprehensive examination of the upper limb nerves in order to provide further evidence in defining and locating a nerve affliction of some kind, e.g. a testing of additional muscles and an assessment of further neurological items. In particular, mechanical nerve trunk allodynia should be looked for [6-8]. With weakness in any of the six muscles, the *isolated* presence of carpal tunnel syndrome, ulnar neuropathy, and radial tunnel syndrome is unlikely. One should recognize the potential presence of double or multiple crush [29,30], both of which, according to our previous studies, seem to be very common phenomena in the studied sample of patients with upper limb symptoms [7].

This study present the outcome of testing of six muscles only but the total examination comprised an assessment of a larger number of neurological items (strength of 16 muscles, sensibility in 7 territories, and nerve trunk mechanosensitivity at 20 locations). The examination started with manual muscle testing followed by sensory testing and finally nerve trunk palpation. In order to avoid a biased assessment of the muscle tests from other findings such as, e.g. abnormal nerve trunk mechanosensitivity, the examiners were supposed to record all findings independently of each other. The low specificity (many weak muscles in non-symptomatic limbs) argues against such bias, which, however, cannot be excluded.

### Limitations

This study is based on the concept that it is possible to test individual muscles in isolation without the interference of other muscles. While it is feasible to test certain muscles in isolation, an entirely correct assessment of individual muscle strength can not be achieved when several muscles participate in a particular movement. This study has aimed to address this challenge by positioning the limb to optimize the function of the muscle to be assessed and at the same time impede the influence of other muscles (Table 1).

The studied six muscles neither represent the Th1 root nor three common upper limb nerve entrapments: ulnar neuropathy at the level of the elbow as well as the wrist, carpal tunnel syndrome and radial tunnel syndrome. The identification of ulnar neuropathy, carpal tunnel syndrome and radial tunnel syndrome requires testing of the strength in the abductor digiti minimi, the abductor pollicis brevis, and the extensor carpi ulnaris muscles, respectively [10]. These conditions may occur in isolation or accompany a more proximal affliction of the radial or median nerves, the brachial plexus, or the roots. The high sensitivity in the examined sample indicates the adequacy of testing six muscles as a screening approach to the upper limb nerves and the rarity of the *isolated* presence of these three common locations of entrapment in a sample of patients referred to a department of occupational medicine.

Symptoms are not necessarily caused by afflictions of the peripheral nerves, but may be caused by upper limb disorders of a non-neurogenous character that should consequently also be examined for. These disorders may occur in isolation, or they may complicate, cause, or accompany upper limb neuropathy. E.g. brachial plexopathy may complicate a shoulder tendonitis; lateral epicondylitis or radio-humeral joint inflammation may affect the adjacent radial or posterior interosseous nerves; carpal tunnel syndrome may develop secondary to increased pressure from inflamed flexor tendons in the carpal tunnel.

Neuropathic symptoms may be located distant to a focal lesion. E.g. elbow or wrist pain may originate from brachial plexopathy or from cervical root compression. Therefore the physical upper limb examination should not to be limited to the symptomatic area but cover the neck and the whole limb.

Clinicians tend to initially interpret upper limb neuropathic pain and dysfunction as carpal tunnel syndrome or cervical root compression, whereas the involvement of the almost one-meter-long intermediate portion of the upper limb nerve-tree is less contemplated and less examined for. Taking into account the frequency by examination of this part of the upper limb nerves of neurologic abnormalities in accordance with infraclavicular brachial plexopathy, median neuropathy, and posterior interosseous neuropathy at elbow level [7] the author regards a limited approach among patients in occupational medicine as a major problem.

### Consequences of the findings

The low specificity (0.45) of an examination limited to six muscles is clearly insufficient for confirmative diagnostics, whereas the high sensitivity may be an argument for using the examination of these muscles as a screening tool for upper limb neuropathy. This screening may be applied in the clinical setting as well as for surveillance of populations, e.g. workers in risk of upper limb neuropathy. The feasibility for use of muscle testing as screening should be studied in exposed populations with varying disease frequency and severity. The relation to symptom status of the outcome of a more extensive blinded examination of neurological items has been demonstrated in a sample of “healthy” and active computer operators [31].

This study does not support the general assumption that altered muscle function cannot be detected in patients with mild nerve compression [32]. In fact, it rather suggests that the failure of including individual muscle strength testing in the physical examination of patients with upper limb disorders – in particular patients, which would otherwise be diagnostically non-classifiable – may have unintended consequences. In the clinical setting, patients may be misinterpreted and mismanaged, or not managed at all. In epidemiological studies insensitive measures of health effects may result in erroneous negative results and consequently missed prevention.

### Upper limb neuropathy as a work-related condition – relation to repetition strain syndrome

This study has not aimed to analyze causation but merely to assess the potentials of a simple physical assessment of the muscle function in a sample of patients referred to a hospital clinic of occupational medicine.

However, a number of reports have dealt with work-related upper limb nerve afflictions [30,32] including brachial plexopathy [23,33]. Werner [34] and Hagert et al. [35] reported rotational loads of the forearm causing radial tunnel syndrome rather than epicondylitis, and Stål et al. described pronator syndrome in a high proportion of female milkers [36]. Recent epidemiological evidence supports that upper limb neuropathy can be work-related as has been shown for, e.g. radial tunnel syndrome [37].

## Conclusions

Manual testing of six upper limb muscles is simple to learn and interpret, and rapid to perform. We have shown that this examination is also reliable, and that the outcome of the examination reflects the symptoms.

In the studied sample of patients referred to a department of occupational medicine, testing of six upper limb muscles has proved to be a highly sensitive approach by being able to identify weakness that may be related to nerve afflictions. Due to limited specificity, however, the examination is not suitable for diagnostic confirmation. It may rather serve as a preliminary screening approach for upper limb neuropathy in individual patients as well as in populations. The examination may be particularly useful when the conventional physical upper limb examination cannot, or cannot fully, explain the patient’s complaints.

If positive, this examination should be followed by further neurological assessment. If negative, the examiner should still consider the potential presence of neuropathic conditions that are not covered by this examination, in particular ulnar neuropathy, radial tunnel syndrome, and carpal tunnel syndrome.

The presented findings may argue that, in the physical examination, clinicians dealing with upper limb disorders should include a manual assessment of muscle strength in six upper limb muscles representing three antagonist pairs: Pectoralis major/posterior deltoid, biceps/triceps, and radial flexor of wrist/short radial extensor of wrist.

## Competing interests

The author declares that he has no competing interests.

## Pre-publication history

The pre-publication history for this paper can be accessed here:

http://www.biomedcentral.com/1471-2377/14/90/prepub
